# Relationship between mucosal healing by tacrolimus and relapse of refractory ulcerative colitis: a retrospective study

**DOI:** 10.1186/s12876-020-01317-9

**Published:** 2020-06-26

**Authors:** Ayumi Ito, Syun Murasugi, Teppei Omori, Shinichi Nakamura, Katsutoshi Tokushige

**Affiliations:** Department of Gastroenterology, Tokyo Women’s Medical University, Kawada-cho 8-1 Shinjuku-ku, Tokyo, 162-8666 Japan

**Keywords:** Ulcerative colitis, Tacrolimus, Mucosal healing

## Abstract

**Background:**

Tacrolimus (TAC) is a powerful remission-inducing drug for refractory ulcerative colitis (UC). However, it is unclear whether mucosal healing (MH) influences relapse after completion of TAC.We investigated whether MH is related to relapse after TAC.

Patients: Among 109 patients treated with TAC, 86 patients achieved clinical remission and 55 of them underwent colonoscopy at the end of TAC. These 55 patients were investigated.

**Methods:**

Patients with MH at the end of TAC were classified into the MH group (*n* = 41), while patients without MH were classified into the non-MH group (*n* = 14). These groups were compared with respect to 1) clinical characteristics before treatment, 2) clinical characteristics on completion of treatment, and 3) the relapse rate and adverse events rates. This is a retrospective study conducted at a single institution.

**Results:**

1) There was a significant difference in baseline age between the two groups before TAC therapy, but there were no significant differences in other clinical characteristics. The NMH group was younger (MH group: 48.1 (23–79) years, NMH group: 36.3 (18–58) years, *P* = 0.007). Endoscopic scores showed significant differences between the 2 groups at the end of TAC. There were also significant differences in the steroid-free rate after 24 weeks (MH group: 85.3%, NMH group 50%, *P* = 0.012). There was no significant difference in the relapse rate between the 2 groups at 100 days after remission, but a significant difference was noted at 300 days (17% vs. 43%), 500 days (17% vs. 75%), and 1000 days (17% vs. 81%) (all *P* < 0.05).

**Conclusions:**

TAC is effective for refractory ulcerative colitis. However, even if clinical remission is achieved, relapse is frequent when colonoscopy shows that MH has not been achieved. It is important to evaluate the mucosal response by colonoscopy on completion of TAC.

## Background

UC is a chronic benign intestinal disease. The prevalence of UC has increased worldwide and is expected to increase further [1]. Surgery may be indicated for severe UC, especially refractory prednisolone-resistant or prednisolone-dependent disease, which is often difficult to treat medically. Nonresponse of UC to steroids leads to surgery 55–85% of the time [2]. However, surgery is associated with various complications [3, 4]. TAC is used as remission induction therapy for refractory UC, and the short-term remission rate achieved with TAC is high [5]. However, the relapse rate at 1 year after induction of remission by TAC is also high, in the range of 20 to 30% [6]. Prevention of relapse after remission has been achieved by TAC, which is an important issue in patients with refractory UC. Reports have recently been published concerning the control of UC relapse by achieving MH [7], but it has not been clarified whether MH influences relapse after TAC therapy. Therefore, this study was performed to investigate the relationship between MH and relapse of UC after TAC.

## Methods

At our department, TAC was administered to 109 patients from April 2016 to December 2018 (mean follow-up period: 819 ± 781 days). TAC was given at 0.025 to 0.075 mg/kg body weight twice daily before breakfast and dinner. Blood samples were collected daily for measurement of TAC levels until the target blood concentration was reached. The TAC dose was adjusted to reach the target trough concentration of 10 to 15 ng/mL blood within two weeks of starting TAC remission induction therapy.

Then, 2 to 3 weeks after the TAC concentration was within the target range, the dose was adjusted again to reach a new lower target concentration of 5 to 10 ng/mL.

**Fig. 1:**
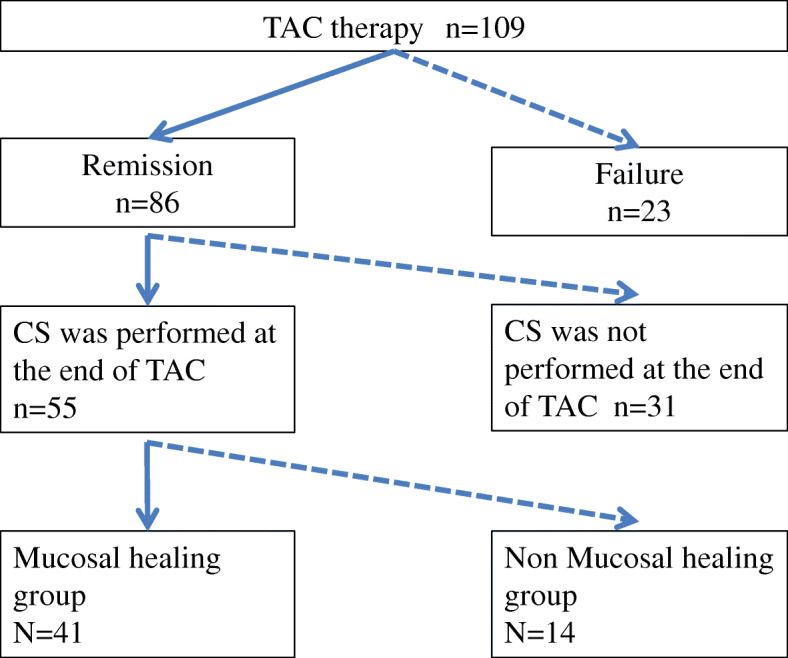
Outline of the mucosal healing group and nonmucosal healing group

Clinical remission was achieved in 86 patients. Colonoscopy was performed at the end of TAC therapy in 55 patients who had agreed to receive colonoscopy (Fig. 1). All patients received steroids before TAC. PSL was administered to all 55 patients before initiation of TAC, so all patients had prednisolone-dependent or prednisolone-resistant refractory UC. Prior to TAC, 5 patients had used biologics.

Endoscopic evaluation was performed to determine the Mayo score and the UCEIS score [8, 9]. Before steroid administration, 42 patients underwent endoscopy (MH group: *n* = 33, NMH group: *n* = 9). MH was defined as a Mayo score of 0 or 1 [7]. Patients who achieved MH at the end of TAC were classified into the MH group (group: *n* = 41), while patients who did not were classified into the non-MH group (group: *n* = 14). The Lichtiger score was determined as the clinical activity index (CAI) [10]. The MH and NMH groups were compared with respect to the following three factors: 1) clinical characteristics before PSL, CAI, hemoglobin, albumin, CRP (C-reactive protein), endoscopic scores (Mayo and UCEIS) and TAC (sex, age, duration of UC, site of UC, PSL responsiveness (dependent/resistant), CAI, hemoglobin, albumin, CRP, endoscopic scores (Mayo and UCEIS), and time to reach the target trough level of TAC); 2) clinical characteristics at the end of TAC (CAI, hemoglobin, albumin, CRP, endoscopic scores (Mayo and UCEIS), total PSL dose during hospitalization, duration of TAC, frequency of combined azathioprine (AZA) therapy, and steroid-free rate after 24 weeks), and 3) the relapse rate at 100 (MH group: *n* = 39, NMH group: *n* = 15), 300 (MH group: *n* = 35, NMH group: *n* = 11), 500 (MH group: *n* = 32, NMH group: *n* = 3), and 1000 days (MH group: *n* = 16, NMH group: *n* = 3) after achieving remission. Remission was defined as a CAI ≤4 at 4 weeks or longer after initiation of remission induction therapy. The total PSL dose during hospitalization was the amount of PSL used until a clinical remission and discharge. Surgery was not required as a result of induction. Relapse was defined as the need for high-dose intravenous steroid therapy, switching to a biologic, re-administration of TAC, or re-administration of TAC at a higher dose (target trough level ≥ 10 ng/dL) to induce remission again.

Adverse events were defined as any undesired or unintended illness or signs thereof (including abnormal laboratory values) occurring in subjects receiving TAC.

### Statistical analysis

The results are expressed as the number of patients or as the mean ± standard deviation. The Wilcoxon test was used for comparisons between the 2 groups, and differences were considered to be significant at *P* < 0.05. JMP Pro12 (Statistical Discover, SAS) was used for all analyses.

## Results

### Clinical characteristics

The clinical characteristics of the MH and NMH groups are summarized in Table 1.

**Table 1:** Comparision between the mucosal healing group (MH Group) and non-mucosal healing group (NMH Group) before treatment

	MH Group *n* = 41	NMH Group *n* = 14	*p*-value
Data before PSL administration			
CAI	13.3 (9–19)	13.1 (10–17)	0.493
Hb (g/dl)	11 (7.5–12.9)	12.6 (7.9–15.1)	0.19
Alb (g/dl)	3.4 (1.9–4.4)	3.4 (2–4.6)	0.889
CRP (mg/dl)	3.9 (0.2–22.6)	2.9 (0.04–11.9)	0.636
Mayo score^a^	2.9 (2–3)	3 (3)	0.341
UCEIS score^a^	6.6 (4–8)	7.4 (6–8)	0.236
Data before TAC administration			
CAI	12.9 (7–19)	13.4 (11–17)	0.593
Hb (g/dl)	12 (7.5–14.9)	12.9 (8.1–15.1)	0.191
Alb (g/dl)	3.3 (1.7–4.4)	3.3 (2–4.6)	0.838
CRP (mg/dl)	3.5 (0.04–24.1)	2.6 (0.04–10.4)	0.525
Mayo score	2.9 (2–3)	3 (3)	0.306
UCEIS score	6.7 (4–8)	7.2 (6–8)	0.136
Time to achieve the target TAC trough level (days)	3.1 (1–10)	4.5 (1–9)	0.07

Data are expressed as the mean ± standard deviation or the number of patients

*Alb* albumin, *CRP* C-reactive protein, *Hb* hemoglobin, *Mayo scar* Mayo endoscopic score, *ns* not significant, *PSL* prednisolone, *TAC* tacrolimus, *TNFα, UCEIS* ulcerative colitis endoscopic index of severity

^a^Colonoscopy before PSL administration was performed in 33 patients in the MH group and 9 patients in the NMH group

Before PSL administration, there was no significant difference between the two groups in CAI MH group: 13.3 (9–19), NMH group: 13.1 (10–17), *P* = 0.493, hemoglobin MH group: 11 (7.5–12.9), NMH group: 12.6 (7.9–15.1) g/dL, *P* = 0.19, albumin MH group: 3.4 (1.9–4.4), NMH group: 3.4 (2–4.6) g/dL, *P* = 0.889, CRP MH group: 3.9 (0.2–22.6), NMH group: 2.9 (0.04–11.9) mg/dL, *P* = 0.636, or either endoscopic score (Mayo: MH group: 2.9 (2, 3), NMH group: 3 (3), *P* = 0.341; UCEIS MH group: 6.6 (4–8), NMH group: 7.4 (6–8), *P* = 0.236) (Table 1).

The age at initiation of treatment showed a significant difference between the 2 groups (MH group: 48.1 (23–79) years, NMH group: 36.3 (18–58) years, *P* = 0.007), but there were no significant differences of other factors (sex M/F MH group: 20/21, NMH group: 9/5, *P* = 0.396; duration of UC MH group: 9.4 (0–33) years, NMH group: 9.0 (0–26) years, *P* = 0.88; site of UC left colon/total colon MH group: 25/16, NMH group: 8/6, *P* = 0.8; steroid responsiveness (dependent/resistant) MH group: 18/23, NMH group: 6/8, *P* = 0.889; CAI: MH group: 12.9 (7–19), NMH group: 13.4 (11–17), *P* = 0.593; hemoglobin MH group: 12 (7.5–14.9), NMH group: 12.9 (8.1–15.1) g/dL, *P* = 0.191; albumin MH group: 3.3 (1.7–4.4), NMH group: 3.3 (2–4.6) g/dL, *P* = 0.838; CRP MH group: 3.5 (0.04–24.1), NMH group: 2.6 (0.04–10.4) mg/dL, *P* = 0.525; endoscopic scores Mayo: MH group: 2.9 (2, 3), NMH group: 3 (3), *P* = 0.306; UCEIS MH group: 6.7 (4–8), NMH group: 7.2 (6–8), *P* = 0.136); and time to achieve the target trough level of TAC MH group: 3.1 (1–10), NMH group: 4.5 (1–9) days, *P* = 0.07) (Table 1).

### Clinical characteristics on completion of TAC

At the end of TAC, significant differences in the endoscopic scores were noted between the 2 groups (Mayo score MH group: 0.6 (0–1), NMH group: 2.2 (2, 3); UCEIS score MH group: 1.1 (0–4), NMH group: 4.4 (4, 5), both *P* = 0.001). There was also a significant difference in the duration of TAC (MH group: 297.6 (58–922) days, NMH group: 136.9 (70–373) days, *P* = 0.014) and the steroid-free rate after 24 weeks (MH group: 85.3%, NMH group 50%, *P* = 0.012). However, there were no significant differences between the 2 groups with regard to CAI (MH group: 2.8 (1–4), NMH group: 2.7 (1–4), *P* = 0.741), hemoglobin (MH group: 11.7 (9.6–15.6), NMH group: 13.2 (11–15.8) g/dL, *P* = 0.098), albumin (MH group: 3.8 (3–4.7), NMH group: 3.8 (2.9–4.6) g/dL, *P* = 0.703), CRP (MH group: 0.12 (0.04–1.15), NMH group: 0.15 (0.03–1.03) mg/dL, *P* = 0.665), total dose of PSL up to remission MH group: 685 (330–1690), NMH group: 755 (335–1322) mg, *P* = 0.501), or use of AZA (MH group: 34/7, NMH group: 11/3, *P* = 0.715) (Table 2).

**Table 2:** Comparision between mucosal healing group (MH Group) and non-mucosal healing group (NMH Group) at the end of TAC

	MH Group *n* = 41	NMH Group *n* = 14	*p*-value
TAC at the end of data			
CAI	2.8 (1–4)	2.7 (1–4)	0.741
Hb (g/dl)	11.7 (9.6–15.6)	13.2 (11–15.8)	0.098
Alb (g/dl)	3.8 (3–4.7)	3.8 (2.9–4.6)	0.703
CRP (mg/dl)	0.12 (0.04–1.15)	0.15 (0.03–1.03)	0.665
Mayo score	0.63 (0–1)	2.21 (2–3)	0.001
UCEIS score	1.19 (0–4)	4.42 (4–5)	0.001
Total prednisolone dose during hospitalization (mg)	685 (330–1690)	755 (335–1322)	0.501
Duration of TAC (days)	297.6 (58–922)	136.9 (70–373)	0.014
Combined use of AZA (yes / no)	34/7	11/3	0.715
Steroid free rate at 24 weeks n (%)	35 (85.3%)	7 (50%)	0.012

Data are expressed as the mean ± standard deviation or the number of patients

*AZA* azathioprine

### Relapse rate

The relapse rate at 100 days after induction of remission showed no significant difference between the MH and NMH groups, being 8% in both groups. However, there was a significant difference in the relapse rate between the 2 groups at 300 days (MH group: 17%, NMH group: 43%), 500 days (MH group: 17%, NMH group: 75%), and 1000 days (MH group: 17%, NMH group: 81%) after induction of remission (*P* < 0.05) (Fig. 3, Table 3). Adverse events related to TAC were reported as tremor, renal impairment, headache, and hypomagnesemia in 7, 5, 3, and 3 patients, respectively (Table 4). The prevalence of adverse events was not different between the MH and NMH groups.

**Table 3:** Relapse rate

	MH Group	NMH Group	*p*-value
Relapse rate after 100 days (%)	8	8	N.S.
Relapse rate after 300 days (%)	17	43	*P*<0.05
Relapse rate after 500 days (%)	17	75	*P*<0.05
Relapse rate after 1000 days (%)	17	80	*P*<0.05

**Table 4:** Adverse events from TAC usage

	MH Group *n* = 41	NMH Group *n* = 14
Tremors	5 (12.1%)	2 (14.2%)
Nephropathy	4 (9.7%)	1 (7.1%)
Headache	1 (2.4%)	2 (14.2%)
hypomagnesemia	2 (4.8%)	1 (7.1%)

Data are expressed as the number of patients (percentage)

In no patient was TAC discontinued due to adverse events.

## Discussion

### Clinical characteristics

UC is a chronic inflammatory disease that repeats episodes of relapse and remission.Only 20–30% of patients with UC moderate or more relapse have difficulty in treatment. PSL are used first in moderate or more severe cases [2]. The patients in this study also had moderate or worse UC. The clinical activity index (CAI) before PSL administration was high, and the endoscopy score (Mayo, UCEIS) was high in 42 patients who underwent endoscopy (Table 1). Therefore, PSL was given before TAC in all cases that were steroid-resistant or refractory. PSL is one of the poor prognostic factors for UC. In cases where PSL must be used, repeated relapses require surgery [11]. Therefore, PSL use has been reported to be one of the markers of UC severity. For intractable cases, it is important to prevent relapse after induction and maintain remission.

Various reports have been published on the benefits of achieving MH and this is considered a therapeutic goal for UC, as MH has reduced the rate of relapse [7, 12].

TAC is used to induce the remission of UC. However, the duration of treatment with TAC is not clearly defined in the ECCO Guidelines [2]. In this study, we compared patients treated with TAC who achieved mucosal healing (MH group) or did not achieve mucosal healing (NMH group). Among the clinical characteristics that we investigated, the age at initiation of treatment was significantly lower in the NMH group. In this study, TAC was started at the time of hospital admission and was completed during outpatient follow-up, so oral administration of TAC was managed by the patients themselves after discharge from the hospital. The percentage of young people in the NMH group was high. This may have contributed to reduced oral drug compliance.

It has been reported that compliance with internal medicine is likely to be reduced among young people [13]. TAC blood levels are affected by diet. Therefore, the time for internal use must be adjusted twice a day.

Compliance with oral administration will decline when oral administration becomes more complex due to other drugs [14]. However, this study did not confirm compliance with internal medicine after discharge and did not measure frequent blood troughs.

Therefore, the decline in compliance with oral administration of young people is only speculation. Furthermore, as the clinical symptoms improve, the compliance with oral administration tends to be further reduced [15]. Therefore, it can be suggested that compliance of younger patients decreases after discharge from the hospital, resulting in failure to achieve MH. Other clinical characteristics, particularly the duration of UC, CAI, and endoscopic severity scores before initiation of TAC, were not correlated with MH.

### Clinical characteristics on completion of TAC

**Fig. 2:**
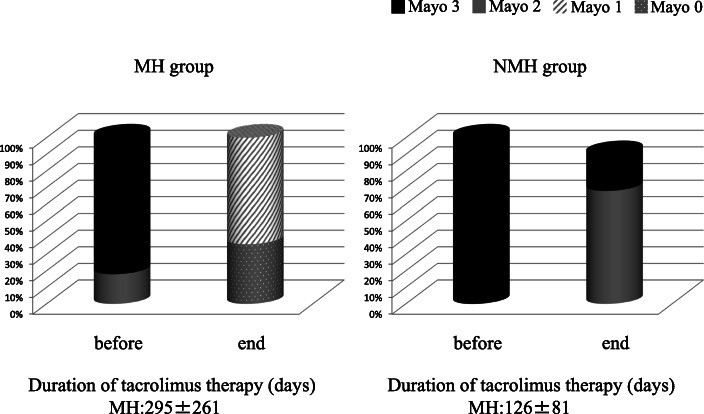
Changes in the endoscopic findings of the MH group and NMH group

Comparison of clinical characteristics between the MH and NMH groups at the end of TAC revealed no significant differences in CAI, hemoglobin, albumin, or CRP. At the end of treatment, detailed data about clinical characteristics and laboratory parameters were obtained from both groups. However, there were no significant differences in clinical or laboratory characteristics between the 2 groups, suggesting that it is difficult to predict the achievement of MH based on these factors. By definition, there were significant differences in endoscopic findings between the MH and NMH groups (Table 2, Fig. 2). Importantly, the duration of TAC was markedly different between the MH and NMH groups, being significantly longer in the MH group (Table 2).

There are no rules regarding the duration of TAC administration. Therefore, the duration of TAC administration varied from physician to attendant, resulting in a variable duration of administration.

This suggests the possibility that MH is more likely to be obtained by long-term treatment with TAC. Therefore, it may be necessary to continue TAC after clinical remission if MH has not been achieved. There was no significant difference in the combination of AZA between the MH group and NMH group. Therefore, whether AZA contributed to the promotion of MH was unclear in this study. The steroid-free rate at 24 weeks showed a significant difference between the 2 groups. PSL are generally the first-line treatment for moderate/severe UC [2, 16]. If there is no response to PSL, switching therapy or the addition of other drugs is required. When a response is noted, the PSL dosage should be tapered or discontinued as soon as possible because these drugs have no remission-maintaining effect and prolonged use may cause steroid dependence, leading to intractability of UC [2, 16–18, 19]. In this study, TAC was not used in the NMH group for long enough. Therefore, MH could not be achieved in the NMH group, and steroid-free treatment could not be achieved. TAC must be used for a sufficient period to be steroid free.

Prolonged use of steroids may also cause a wide variety of adverse events [18]. Accordingly, achievement of a steroid-free status in UC patients is important to prevent intractability of the disease and steroid-related adverse events [2, 20].

### Relapse rate

**Fig. 3:**
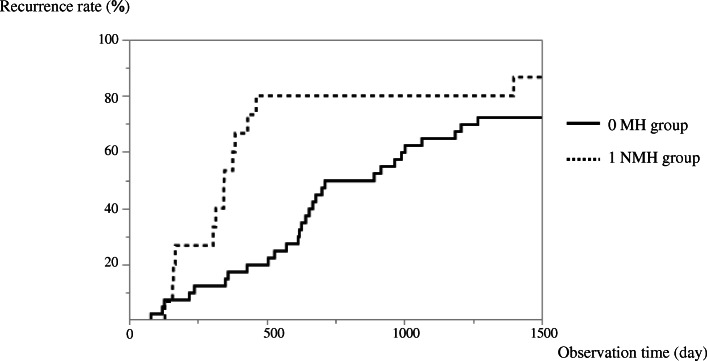
Relapse rate after achieving remission

At 100 days after induction of remission, the relapse rate was the same in the MH and NMH groups (MH group: 8%, NMH group: 8%), but the relapse rate showed a significant difference between the two groups at 300 days (MH group: 17%, NMH group: 43%), 500 days (MH group: 17%, NMH group: 75%), and 1000 days (MH group: 17%, NMH group: 81%) (*P* < 0.05) (Fig. 3, Table 3). That is the relapse rate was lower from 300 days on in the MH group. It has been reported that MH affects the recurrence and maintenance of remission [11]. Achieving MH in infliximab studies has been reported to contribute to the maintenance of remission. Achieving MH is considered to be a target for UC treatment. This study focused on achieving MH with TAC [12]. Unlikely previous reports, we focused on the achievement of MH by TAC in this study.

There are few reports examining the relationship with MH in refractory cases using TAC. Miyoshi et al. reported that colonoscopy results 3 months after TAC were associated with later relapse [21]. This study also reports that MH was involved in maintaining remission. However, our study differs from Miyoshi’s report in the period of use of TAC. TAC was used until MH was achieved to prevent relapse.

The importance of preventing relapse differs between patients with refractory or moderate/severe UC and patients with mild UC. Refractory or relapsing moderate/severe UC results in frequent hospital attendance, admission, and intensified drug therapy, which have a detrimental impact on the quality of life [22, 23].

Furthermore, surgery is often required to manage patients with refractory or relapsing moderate/severe UC [4]. Therefore, maintenance of remission is very important for refractory or relapsing moderate/severe UC.

As adverse events related to TAC therapy, tremor (7 patients), renal impairment (5 patients), headache (3 patients), and hypomagnesemia (3 patients) were observed in the present study (Table 4). All of these events improved after dosage reduction of TAC. Renal impairment was improved by the addition of fluids and TAC could be continued [24, 25].

A strength of this paper is that the total amount of PSL until remission (mg), the number of days until the TAC trough was achieved (day), and the TAC administration period are examined. There were several reports on TAC and MH. However, there is no report on these points. This suggests that this report may serve as a standard for treatment in clinical practice.

Unfortunately, this study did not use calprotectin to assess MH. It has been reported that calprotectin is simple and is an excellent biomarker for predicting MH and relapse. In the future, it is necessary to consider additional factors such as this [26].

The limitations of this study included its retrospective design and its collection of data from a single center, which could have resulted in bias. To confirm our findings, it will be necessary to perform a prospective multicenter study in a larger number of patients.

## Conclusions

TAC is effective for refractory UC, but relapse can occur after clinical induction of remission if colonoscopy shows that MH has not been achieved. Colonoscopy should always be performed to evaluate the mucosal response at completion of TAC. We suggest continuing TAC for long-term MH.

## Data Availability

The datasets during and/or analysed during the current study available from the corresponding author on reasonable request.

## Abbreviations

AZA: Azathioprin
CAI: Clinical active index
CRP: C-reactive protein
MH: Mucosal healing
NMH: Nonmucosal healing
TAC: Tacrolimus
PSL: Prednisolone
UC: Ulcerative colitis
UCEIS: Ulcerative colitis endoscopic index of severity
